# SpliceHetero: An information theoretic approach for measuring spliceomic intratumor heterogeneity from bulk tumor RNA-seq

**DOI:** 10.1371/journal.pone.0223520

**Published:** 2019-10-23

**Authors:** Minsu Kim, Sangseon Lee, Sangsoo Lim, Sun Kim

**Affiliations:** 1 Department of Computer Science and Engineering, Seoul National University, Seoul, 08826, Korea; 2 Interdisciplinary Program in Bioinformatics, Seoul National University, Seoul, 08826, Korea; 3 Bioinformatics Institute, Seoul National University, Seoul, 08826, Korea; CHOC Children’s Hospital - UC Irvine, UNITED STATES

## Abstract

**Motivation:**

Intratumor heterogeneity (ITH) represents the diversity of cell populations that make up cancer tissue. The level of ITH in a tumor is usually measured by a genomic variation profile, such as copy number variation and somatic mutation. However, a recent study has identified ITH at the transcriptome level and suggested that ITH at gene expression levels is useful for predicting prognosis. Measuring ITH levels at the spliceome level is a natural extension. There are serious technical challenges in measuring spliceomic ITH (sITH) from bulk tumor RNA sequencing (RNA-seq) due to the complex splicing patterns.

**Results:**

We propose an information-theoretic method to measure the sITH of bulk tumors to overcome the above challenges. This method has been extensively tested in experiments using synthetic data, xenograft tumor data, and TCGA pan-cancer data. As a result, we showed that sITH is closely related to cancer progression and clonal heterogeneity, along with clinically significant features such as cancer stage, survival outcome and PAM50 subtype. As far as we know, it is the first study to define ITH at the spliceome level. This method can greatly improve the understanding of cancer spliceome and has great potential as a diagnostic and prognostic tool.

## Introduction

Intratumor heterogeneity (ITH) represents the diversity of cell populations that make up cancer tissue [1]. This results from a subclone diversification process during cancer progression, which is considered a form of Darwinian evolutionary process [2]. The level of ITH reflects the genetic diversity of bulk tumors, which generally has a negative impact on prognosis. An explanation for this trend is that the genetic diversity provided by ITH can be an accelerator of somatic cell evolution that helps cancer cells acquire a malignant phenotype [3, 4, 5, 6]. Studies have been published that measure the level of ITH in bulk tumors in the whole genome level [7, 8, 9].

In a recent study by Morris et al. [10], The ITH of each cancer sample was calculated using genomic features such as copy number variation (CNV) and somatic mutation. Then, the relationship between the ITH of each cancer sample and various clinical characteristics was tested. They concluded that the level of ITH in each cancer sample was significantly associated with the molecular, pathological, and clinical characteristics including prognosis. Other studies are supporting the results of Morris et al. [11, 12].

### Related works

ITH can be deduced using molecular profiles extracted from genomic, epigenomic and transcriptomic data such as whole-genome sequencing (WGS), CNVs, bisulfite-seq, and RNA-seq data. Approaches using each domain have been used to assess the level of ITH in cancer tissues and to identify molecular features associated with tumor evolution (Table 1). For example, two ITH studies using genomic variation have revealed somatic mutations that are closely related to tumor evolution in various types of cancer [13, 14]. Methylomic and transcriptomic (gene-expression) methods for measuring ITH in bulk tumors were developed and identified important molecular features [15, 16].

**Table 1:** Description of each approach using various molecular domains.

Domain^1^	Variation^2^	Method^3^	Findings^4^
Genomic	CNVs Somatic mutations	Mathematical modeling	Suggesting CDK12 as a candidate TSG in ovarian carcinoma [13]
Methylomic	Methylation	Mathematical modeling	Association between mutations in chromatin modifiers like SMARCA4, BAP and methylomic ITH [16]
Transcriptomic	Gene expression	Information theory	Suggesting that cell cycle related pathways have significant contribution to increasing heterogeneity on the network during clonal evolution [15]
Spliceomic	Alternative splicing	None	None

^1^ The Domains column represents the domain data used in each approach.

^2^ The Variation column represents the genetic variation that each approach uses to construct the model.

^3^ The Method column indicates the type of algorithm each approach uses to build the model.

^4^ The Findings column explains the results revealed using each approach.

Genome-level ITH has been extensively studied using bulk tumor sequencing data. ABSOLUTE [13] is a genomic ITH (gITH) model that uses somatic mutations and CNV profiles of bulk tumors to infer ITH. ABSOLUTE estimated the optimal values of cancer purity and ploidy using a linear programming technique and then estimated the subclonal genome fractions (ie, ITH). A slightly different approach was used in PyClone [14]. PyClone used the Bayesian model to define the generative relationship between the number of subclones and the observed genomic variation and then used the Bayesian clustering algorithm to select the optimal number of subclones that best fit the observed data.

Recently, an ITH model using a methylation profile was developed. This model, proposed by Mazor et al. [16], used a mathematical modeling approach similar to the genomic data based model. DNA methylation does not alter the DNA sequence but is linked to genomic DNA. Thus, the DNA methylation pattern has similar characteristics to the genomic variants. For example, when bisulfite-seq is used, methylation base detection is similar to somatic mutation detection.

A transcriptome-level ITH model was recently developed [15]. They used information theory to estimate ITH in bulk tumors. They proposed an interesting idea to consider ITH as the difference in gene expression distribution between normal tissue and bulk tumors. They first used a curated database of molecular pathways, such as the KEGG database [17], to construct a template network and construct a probability distribution for each pathway. The divergence between normal tissue and bulk tumor samples is then calculated by the average Jensen-Shannon Divergence (JSD) of each probability distribution for each pathway. This divergence was considered to be transcriptomic ITH (tITH) for each sample and was found to be related to clonal evolution and prognostic features.

### Motivation

Splicing variations have been regarded as important cancer drivers in various types of cancer [18, 19, 20, 21]. In 2018, Kahles et al. performed a pan-cancer level study that investigates the impacts of splicing variations on cancer progression [22]. They performed a systematic analysis of alternative splicing landscape across 8,705 cancer patients and found that many tumors contain numerous novel splicing junctions, which is not typically found in normal samples. And these novel junctions were identified as a class of potential neo-antigens. This means that the splicing variant of cancer cells can be an important factor in the evolution of the tumor. Other studies support the idea [20, 23].

The presence of intercellular spliceomic differences has also been proposed by numerous studies [24, 25]. A recent single-cell study showed that there is a clear difference in the use of isoforms in bone marrow-derived dendritic cells [26]. The clinical effect of spliceomic ITH (ie, sITH) has not been thoroughly studied because a systematic sITH model has not yet been developed.

Since Park et al. [15] proposed a pioneering model of ITH at the RNA level, the spliceomic ITH (ie, sITH) is naturally defined by adopting their method [15]. However, there are serious technical difficulties in applying their methods to sITH. First, the tITH model by Park et al. [15] requires a template network to generate probability distributions that are not available in our case. Also, a recent study has reported the widespread intron retention of cancer cells [27], suggesting that the isoform of cancer cells is very complex and not yet characterized. This means that a significant amount of unexpected splice junctions can be found in the cancer sample [28]. To handle these unexpected splice junctions, a transcriptome assembly is required to account for previously unknown isoforms. However, prevalent splice-site mutations [29] and short sequence reads in RNA-seq make it difficult to perform transcriptome assembly. Therefore, a new model is needed to avoid this difficulty and to measure sITH in bulk tumors.

The flow of subsequent manuscripts is as follows: 1) definition of the proposed model, 2) basic verification of the validity of the model, 3) performance verification of the model using synthetic data, 4) performance verification using xenograft tumor data, and 5) performance verification using TCGA pan-cancer data.

## Materials and methods

In this study, we used bulk-tumor RNA-seq data to measure sITH. It is a technique that combines bulk sampling with short-read sequencing. A possible alternative for each part is single-cell analysis and single-molecule real-time sequencing (SMRT-seq). Single-cell analysis has been improved in terms of stability and efficiency and has been used in many biological studies. This technique is very useful for studying ITH because it provides a molecular profile of every single cell in bulk tumors [30]. However, due to patient-to-patient heterogeneity, extensive analysis of a large group of patients is required to produce a reproducible cancer model. Thus, a single cell approach is not feasible in this case. Another technology, SMRT-seq, has received much attention because of its long read length and the advantage of being free from bias induced by cDNA amplification. However, sequencing errors in SMRT-seq are still a problem [31] and production costs are still very high. Currently, major cancer consortia such as TCGA produce only bulk tumor RNA-seq data. It is therefore difficult to obtain data with adequate clinical information using the SMRT-seq platform or single-cell platform. Thus, this study focused primarily on bulk-tumor RNA-seq.

### Definition of the proposed model

**Fig 1:**
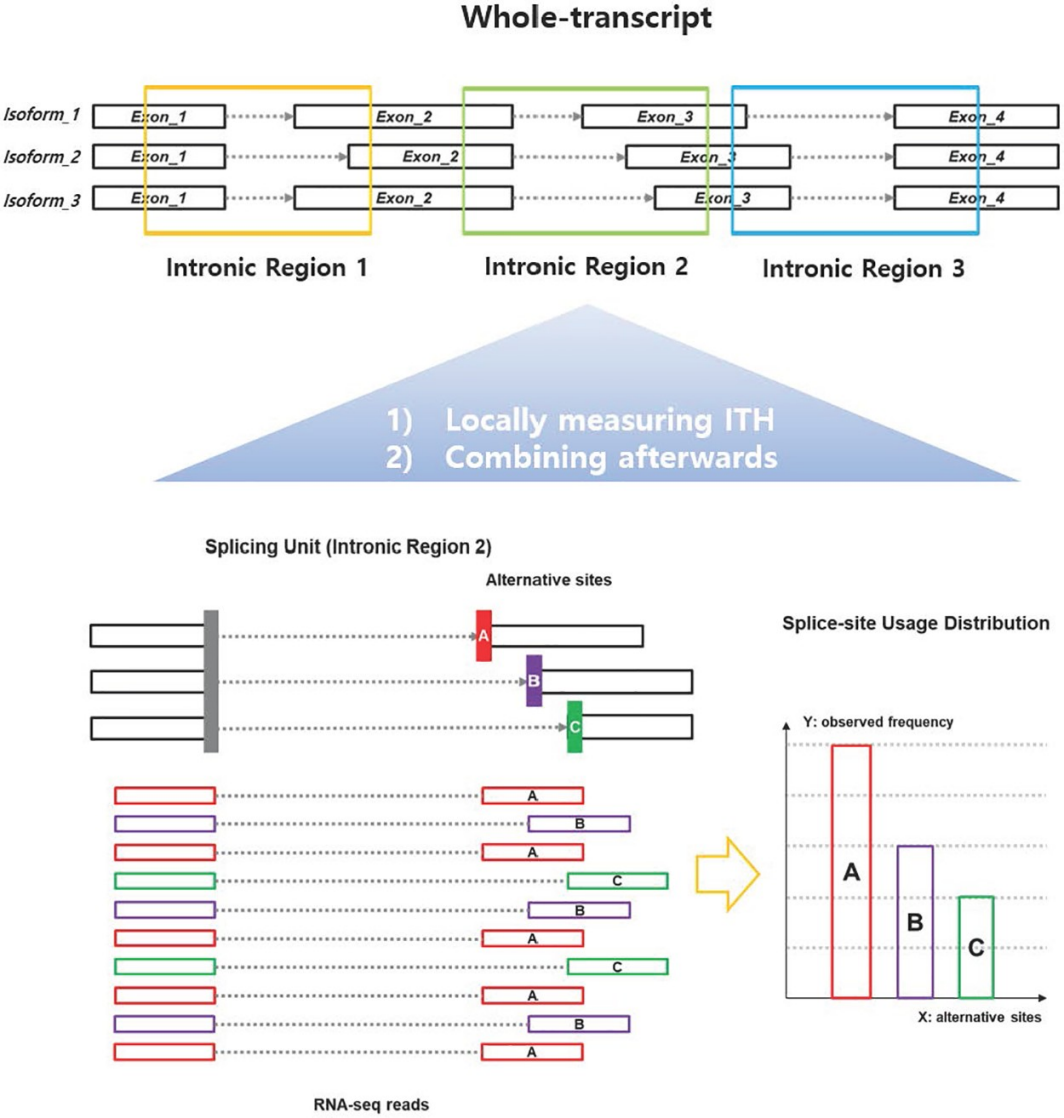
Illustration of an intronic splicing unit. An intronic splicing unit is defined as a set of splicing events that share a common splicing site (i.e., donor or receiver) in the intronic domain. Each intronic splicing unit consists of an isoform usage distribution of each sample in each locus. Here, the splice-site usage distribution is calculated by the number of RNA-seq reads that support each alternative splice-site (shown in red, purple, and green in the figure).

As discussed in the previous section, transcript assembly in cancer is very difficult due to complex splicing patterns, noncanonical splice sites, and short-length sequence reads. So we decided to use a local analysis approach to avoid transcriptome assembly. Here, each RNA-seq read that supports each splicing event is collected and grouped by each intron region. (Fig 1). Spliced aligners such as RNA-STAR [32] align RNA reads with reference genome sequences and output mapped positions on chromosomes. Because RNA-seq reads are originated from mature mRNA transcripts, the spliced region remains a gap in the final alignment. The aligner collects the spliced gaps and organizes them into splice sites (i.e., the ends of the intron). As a result, the aligner lists the position of each splice site on the chromosome observed in a given RNA-seq and the number of supporting reads. The list of splice junctions extracted from the RNA-seq of each bulk tumor is the input data to construct our model (Fig 1).

Then we defined a local unit called an *intronic splicing unit* or *splicing unit*, which is defined as a collection of splicing events for each intron (Fig 1). In this scheme, splice junctions sharing a common splice site are grouped into a single unit. As in Fig 1, if three splice junctions are sharing a common splice site upstream and three alternate sites downstream, a splicing unit S consisting of A, B, and C can be defined. Where the input variable is defined by the junction count of A, B and C (i.e. *CNT*_*S*_ = (5, 3, 2), Eq 1). By dividing the sum of the total observations we can get the probability distribution for S (i.e. *P*_*S*_ = (0.5, 0.3, 0.2), Eq 1).

$$\begin{array}{c} P_{k}\left(i\right)=\frac{CNT_{k}\left(i\right)}{\sum_{j=1}^{N_{k}}CNT_{k}\left(j\right)} \end{array}$$ (1)

Where *CNT*_*k*_(*i*) represents the number of RNA-seq reads that support *i*-th alternative splice site in *k*-th splicing unit. *P*_*k*_(*i*) is the fraction of RNA-seq reads that support the *i*-th splice site of the *k*-th splicing unit. *N*_*k*_ is the total number of alternative sites in *k*-th splicing unit.

Normal tissues are also known to have heterogeneity in the use of isoforms between cells [26]. To deal with this, we defined the spliceome ITH (i.e., sITH) as the distance from the normal tissue sample to the bulk tumor sample. By doing so, the model is expected to eliminate the innate heterogeneity that exists in normal tissues, leaving only the perturbations that occur during cancer progression. We used the Jensen-Shannon Divergence (JSD), which is defined by averaging bidirectional Kullback-Leibler Divergences (KLDs) from the introduced intermediate data points (Eq 2) [33, 34]. Then JSD gets the symmetric property and the metric value is limited from 0 to 1 (if you are using a base 2 log). JSD has been used in bioinformatics studies for its symmetric property [35, 36]. We defined input variables representing the distribution of isoform usage for each sample of each locus as a JSD-computable form (Eq 1).

JSD can be calculated for each intronic region (Eq 2). Because each input variable is intended to reflect the use of the splice site at each intronic region, the JSD between the two samples indicates how much the two samples differ in their use of the splice site in that intronic region. This distance is scaled from 0 to 1. Where 0 means that the splice site usage pattern is the same and 1 is completely different. After calculating the JSD for each splicing unit, a single indicator representing the ITH of entire spliceome is calculated by averaging the JSD of all units (Eq 5). We named this indicator as spliceomic intratumor heterogeneity (or sITH). The detailed calculation procedure is as follows.

$$\begin{array}{c} JSD\left(P_{k},Q_{k}\right)=\frac{1}{2}(KLD(P_{k}\left|\right|M_{k})+KLD(Q_{k}\left|\right|M_{k})) \end{array}$$ (2)

$$\begin{array}{c} KLD(P_{k}\left|\right|M_{k})=-\sum_{i=1}^{N_{k}}P_{k}\left(i\right)log\frac{P_{k}\left(i\right)}{M_{k}\left(i\right)} \end{array}$$ (3)

$$\begin{array}{c} M_{k}\left(i\right)=\frac{1}{2}\left(P_{k}\left(i\right)+Q_{k}\left(i\right)\right) \end{array}$$ (4)

*JSD*(*P*_*k*_, *Q*_*k*_) represents the Jensen-Shannon divergence between the two distributions *P*_*k*_ and *Q*_*k*_, where *P*_*k*_ and *Q*_*k*_ denote the splice-site usage distribution of the *k*-th splicing unit in samples P and Q, respectively. Note that *P*_*k*_ and *Q*_*k*_ are defined in Eq 1. *M*_*k*_ represents the intermediate distribution introduced between two distributions *P*_*k*_ and *Q*_*k*_ designed to calculate bi-directional KLD (Eq 4). *KLD*(*P*_*k*_‖*M*_*k*_) represents the Kullback-Leibler divergence of the distribution *P*_*k*_ from *M*_*k*_. Ther might be a case that two samples have different sets of splice sites. In that case, the pseudo-count is added to the splice site, which is not found in one sample, where the pseudo-count is calculated to be 1/100 of the total number of reads in the corresponding splicing unit.

$$\begin{array}{c} sITH\left(P,Q\right)=\frac{1}{L}\sum_{k=1}^{L}JSD\left(P_{k},Q_{k}\right) \end{array}$$ (5)

**Fig 2:**
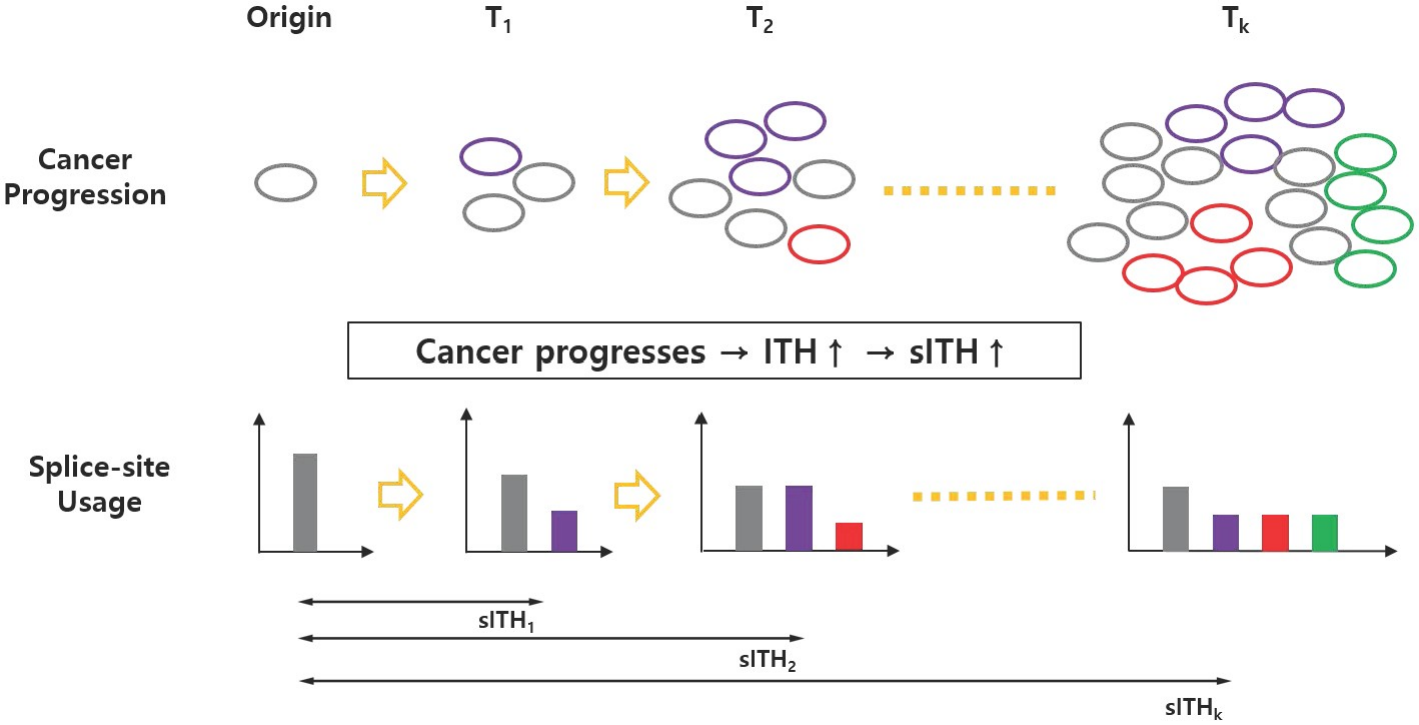
An illustration of how cancer progression affects splice-site usage distribution and spliceomic ITH. Clonal heterogeneity increases as a result of cancer progression, which changes the distribution of splice site use in bulk tumors. The sITH is also designed to increase accordingly.

*sITH*(*P*, *Q*) represents the increased sITH of a sample *P* from the origin sample *Q* to be compared. In the actual case, the target sample P corresponds to a bulk tumor sample, and the origin sample Q corresponds to a normal sample. In this case, *sITH*(*P*, *Q*) may be called sITH of sample P for convenience (Fig 2). *L* represents the total number of splicing units (usually 20∼30 thousands units found in human cancer tissue). Here, the two samples to be compared are pre-processed using the pseudo-counting described above to have the same number of splicing units for the compatibility issue. Therefore, the *i*-th splicing unit of samples *P* and *Q* represents the same intronic region.

### Basic verification of the validity of the model

Before the full-scale analysis, we validated the local analysis approach described above. The key question is whether ITH measured in intron units can replace ITH measured in whole-transcript units. To do this, we prepared a simple experiment using synthetic data. First, 10 genes are randomly selected based on the NCBI RefSeq gene annotation [37]. RNA-seq samples with arbitrary isoform blending ratios were synthesized for these 10 selected genes. To simplify the problem, we simulated heterogeneity by selecting the longest isoform for each gene as the representative transcript and mixing the remaining isoforms at an arbitrary rate. Simulation of RNA-seq data was performed using WgSim, a well known NGS data synthesis tool (CMD: wgsim -e0 -r0 -R0 -X0 -S0 -A1 -d 500 -s 5) [38].

The whole-transcript ITH (ie *ITH*_*transcript*_) of these RNA-seq samples was defined using Shannon’s entropy as Eq 6, which was proposed by Graf et al. [39]. For the same samples, we define the intronic ITH (ie, *ITH*_*intron*_) as Eq 7. We randomly selected 10 genes 10 times and randomly assigned 100 heterogeneity levels to each gene pool to synthesize a total of 1000 RNA-seq data. The correlation between *ITH*_*transcript*_ and *ITH*_*intron*_ calculated for the 1,000 samples was analyzed. The results are discussed in the “Results” section.

$$\begin{array}{c} ITH_{transcript}=-\frac{1}{10}\sum_{g=1}^{10}\sum_{i=1}^{N_{g}}P_{gi}logP_{gi} \end{array}$$ (6)

*P*_*gi*_ indicates the ratio of the i-th isoform of the g-th gene and implies the sequencing coverage ratio in the context of this analysis. *N*_*g*_ indicates the number of isoforms of the g-th gene.

$$\begin{array}{c} ITH_{intron}=-\frac{1}{L}\sum_{k=1}^{L}\sum_{i=1}^{N_{k}}P_{ki}logP_{ki} \end{array}$$ (7)

*P*_*ki*_ is the selection ratio for the i-th splice site of the k-th intron unit, which means the read count ratio between splicing junctions. *N*_*k*_ is the number of splice sites of the k-th intron unit. *L* indicates the total number of intron units found in the sample.

### Performance verification of the model using synthetic data

The above analysis was to demonstrate the validity of the local analysis approach and this analysis was to verify that the defined sITH model can indeed measure the ITH of bulk-tumors. We experimented with synthetic data by mixing normal breast tissue data with single-cell breast cancer data. The purpose of this experiment was to test how the sITH of the mixed sample changes as the mixing ratio increases. The evaluation process and its results are as follows.

- First, we collected 112 normal breast tissue RNA-seq data from TCGA-BRCA [40]. Then 39 single cell breast cancer data were collected from another study (SRA accession: SRP159204) [41], where the 39 cells were derived from different clones of a single breast tumor.
- Each RNA-seq data was processed to obtain the splicing junctions, and the samples were combined in various combinations. Our goal at this stage was to specify a predefined level of ITH in each of the synthetic mixture samples.
- We randomly selected normal tissue data from 112 normal tissue pools. Single cells were then selected for mixing. Here, the number of cells represents the ITH level (ie, 1 ∼ 39). Selected single cells were mixed in normal tissue at a rate of 1% per cell. The detailed definition of each mixture sample is in Eq 8.
  $$\begin{array}{c} MIX\left(i,j\right)=NT\left(j\right)*\left(1-i/100\right)+\sum_{l=1}^{i}\left(SC\left(l,j\right)/100\right) \end{array}$$ (8)
  The *i* represents the ITH level we want to specify, and *j* represents the *j*-th junction of the mixture sample. *MIX*(*i*, *j*) represents the count of the *j*-th junction of the resulted mixture sample with *i* ITH level. *NT*(*j*) represents the count of *j*-th junction in the selected normal tissue sample. *SC*(*l*, *j*) represents the number of *j*-th junction in the *l*-th selected single-cell cancer sample. To avoid sampling bias we randomly extracted 10 times at each of 39 ITH levels. Thus, a total of 390 mixture samples were synthesized (10 iterations per 39 ITH levels). In conclusion, samples mixed with more single cells are expected to have a larger ITH by design.

We conducted a correlation analysis between the number of single cells mixed and the sITH value measured in the sample, and the results are discussed in detail in the “Results” section.

### Performance verification using xenograft tumor data

The main limitation of the previous synthetic data experiment is the lack of an appropriate evolutionary model in the mixture generation. In this experiment, we used xenograft tumor data (SRA accession: SRP050242) generated by actual clonal evolution [42]. The xenograft mouse model we used is derived from the human breast cancer cell line (MCF10A). Cell lines were treated with *HRAS* transduction before transplantation to enhance malignancy. After the single-cell origin derived from MCF10A-*HRAS* was transplanted into immunocompromised mice, the xenograft tissues were cultured until the tumor completely progressed and metastasized. DNA and RNA samples were collected at various points during the process. Therefore, we can compare the ITH from two different molecular domains (genome and spliceome) as the tumor grows. A total of 10 samples were collected while culturing xenograft tissue. They collected two samples for metastatic tissue and one sample for each of the eight-time points.

First, we calculated the sITH for each xenograft tumor sample, using normal breast tissue samples from TCGA as the origin [40]. At this time, 10 normal tissues are randomly selected to avoid selection bias. This comparative analysis is carried out in the following two perspectives. The first is a comparison of the correlation of sITH with the progression of cancer, which indicates time-point provided by the data producers. The second is a comparison of the number of subclones estimated using genomic data, ie, gITH and sITH. These were also measured by data producers using PyClone. PyClone estimates the subclonal structure of each bulk-tumor based on SNV and somatic mutation data. The gITH used in this experiment was the number of estimated subclones. The comparison results are covered in the “Results” section.

### Performance verification using TCGA pan-cancer data

Unlike xenograft samples from one common ancestral cell, samples from cancer patients were from a diverse population. This patient-to-patient variation in the genetic background can be a confounding factor that can mask actual ITH. It is also unclear whether the origins of the tissue can affect the results of the analysis because only breast cancer tissues were used in previous experiments. To test whether sITH could overcome potential problems and demonstrate its clinical significance, we performed a comprehensive pan-cancer level experiment using the TCGA pan-cancer dataset [43]. Weinstein et al. aggregated the TCGA pan-cancer data used for the analysis and published them through a web page (https://gdc.cancer.gov/about-data/publications/pancanatlas).

We first collected all the RNA-seq data provided by TCGA pan-cancer. Table 2 summarizes the results of the collection. We then measured sITHs for each RNA-seq sample. There are 9,274 RNA-seq samples of primary tumors. Of the 977 samples, sITH can not be calculated because there is no normal tissue sample matched. Therefore, 8,297 samples are available for sITH. The sITH of each bulk tumor was calculated using corresponding normal tissues. For example, BRCA has 1,093 primary tumors and 112 normal tissues (Table 2). In this case, the sITHs of each tumor sample were calculated by averaging sITH for each of the 112 normal tissues.

**Table 2:** A table to summarize the number of samples for each type of cancer used in each comparison.

DISEASE	NT^1^	PT^2^	sITH^3^	gITH^4^	STAGE^5^	SURVIVAL^6^	PAM50^7^
BRCA	112	1,093	1,093	1,020	1,002	325	473
KIPAN	129	889	889	659	632	287	0
GBMLGG	5	669	669	643	0	274	0
STES	46	599	599	558	142	71	0
HNSC	44	520	520	485	422	238	0
LUAD	59	515	515	489	487	211	0
LUSC	51	501	501	465	464	245	0
THCA	59	501	501	446	0	0	0
PRAD	52	497	497	469	0	0	0
BLCA	19	408	408	397	395	210	0
COADREAD	51	379	379	351	257	91	0
LIHC	50	371	371	354	333	155	0
CESC	3	304	304	291	0	99	0
SARC	2	259	259	242	0	0	0
PCPG	3	179	179	160	0	0	0
PAAD	4	178	178	158	66	43	0
UCEC	24	176	176	170	0	0	0
THYM	2	120	120	103	0	0	0
SKCM	1	103	103	103	0	0	0
CHOL	9	36	36	36	36	19	0
OV	0	303	0	0	0	0	0
LAML	0	173	0	0	0	0	0
TGCT	0	150	0	0	0	0	0
MESO	0	87	0	0	0	0	0
UVM	0	80	0	0	0	0	0
ACC	0	79	0	0	0	0	0
UCS	0	57	0	0	0	0	0
DLBC	0	48	0	0	0	0	0
**SUM**	**725(20)**	**9,274(28)**	**8,297(20)**	**7,599(20)**	**4,236(11)**	**2,268(13)**	**473(1)**

^1^ The NT column represents the number of normal tissue samples for each type of cancer.

^2^ The PT column represents the number of primary tumor RNA-seq samples per cancer type.

^3^ The sITH column represents the number of samples for which sITH can be calculated.

^4^ The gITH column represents the number of samples for which both sITH and gITH can be calculated.

^5^ The STAGE column shows the number of samples with cancer stage information among the samples for which sITH and gITH can be calculated.

^6^ The SURVIVAL column represents the number of samples with survival outcome information among the samples for which sITH and gITH can be calculated.

^7^ The PAM50 column shows the number of breast cancer samples with PAM50 label information among the samples for which sITH and gITH can be calculated.

The TCGA pan-cancer data validation consists of four steps: 1) comparison of genomic ITH with sITH, 2) comparison of cancer stage with sITH, 3) comparison of survival outcome with sITH, and 4) comparison of PAM50 subtype and sITH. The comparison results are covered in the “Results” section.

## Results

### Basic verification of the validity of the model

**Fig 3:**
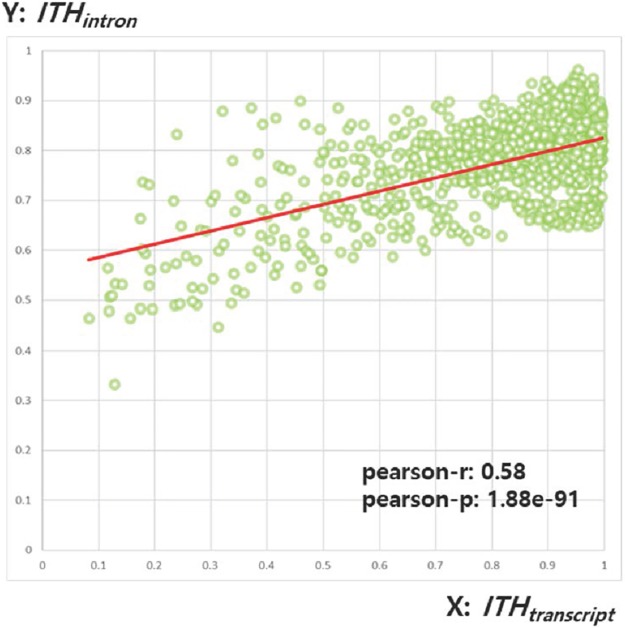
A scatter plot showing the correlation between *ITH*_*transcript*_ and *ITH*_*intron*_. The X axis represents *ITH*_*transcript*_ for each sample and the Y axis represents *ITH*_*intron*_.

The Pearson correlation analysis between *ITH*_*transcript*_ and *ITH*_*intron*_ for 1,000 samples generated with arbitrary heterogeneity levels shows that the two measures have a significant positive correlation (r = 0.58, p = 1.88e-91) (Fig 3). This means that the local analysis approach, which measures ITH in intron units, can effectively reproduce the values measured in whole-transcripts.

### Performance verification of the model using synthetic data

**Fig 4:**
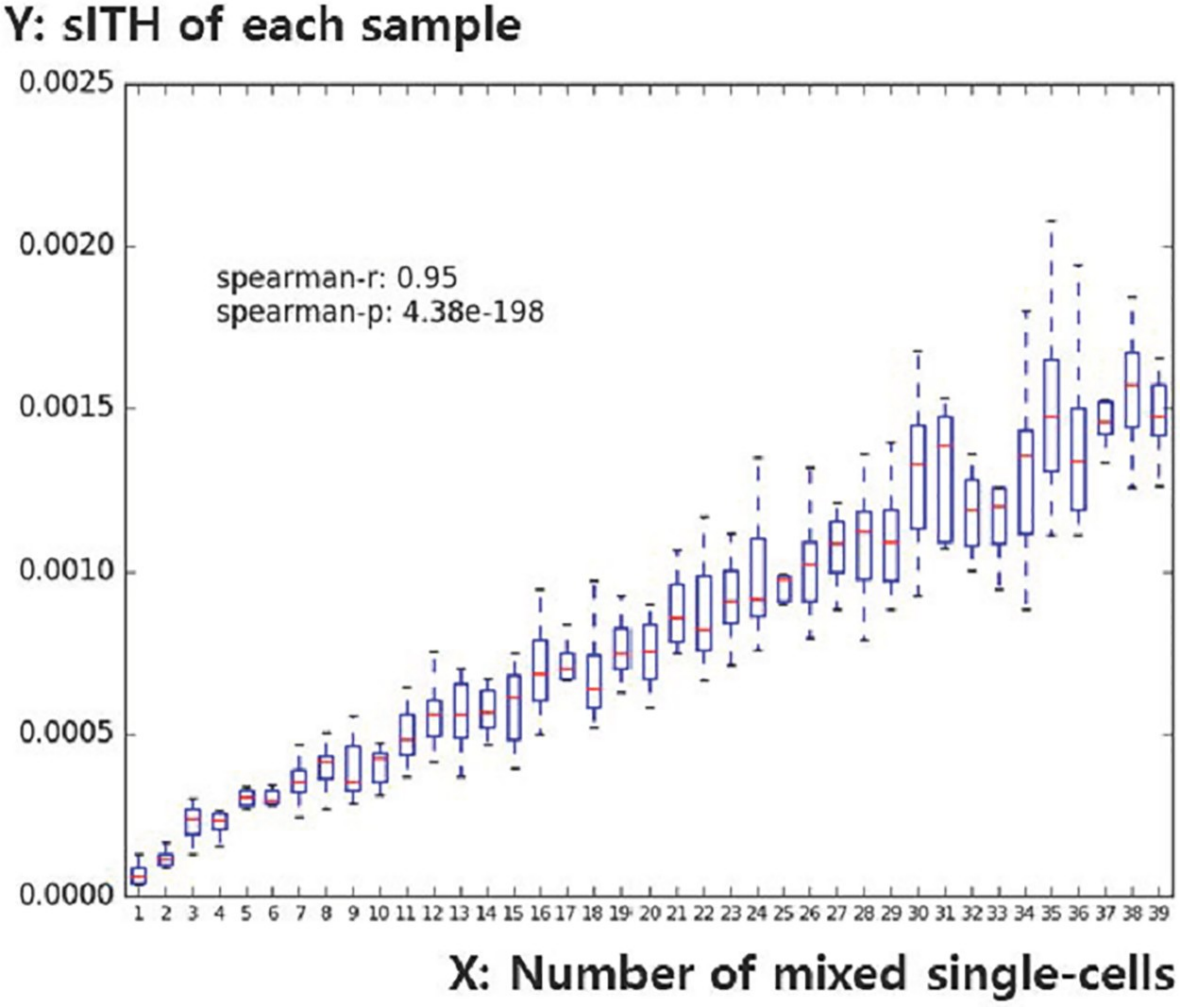
A boxplot to show the association between the number of synthesized single cells and the sITH of synthesized data. The X-axis represents the number of mixed single cells (1∼39). The Y-axis represents the sITH of the sample mixed with the number of single cells specified on the X-axis.

sITH values were measured for samples made by mixing actual single-cell data with actual breast cancer tissues at a defined ratio and compared to the single-cell blending ratio of each sample. Spearman correlation test showed that the ratio of single cells mixed with sITH was positively correlated (r = 0.95, p = 4.38e-198) (Fig 4). This means that the more the single cells are mixed, the higher the sITH is.

One of the problems with this experiment using synthetic data is that an appropriate evolutionary model is not taken into account, which is problematic in the following two points. 1) In this scheme, the ITH level is defined in such a way that different single cells are keep being added to the normal tissue. There is no way to know whether this reflects the actual cancer evolution model. 2) In this scheme, combine single cells is done by simply dividing the junction count, which may be different from the actual experimental results, because there is a limit to the number of reads that can be captured by the RNA-seq technology. These factors may be the cause of the linear and gradual increase of the pattern without the saturation shown in Fig 4 and show the limitations of the experiment using synthetic data.

### Performance verification using xenograft tumor data

**Fig 5:**
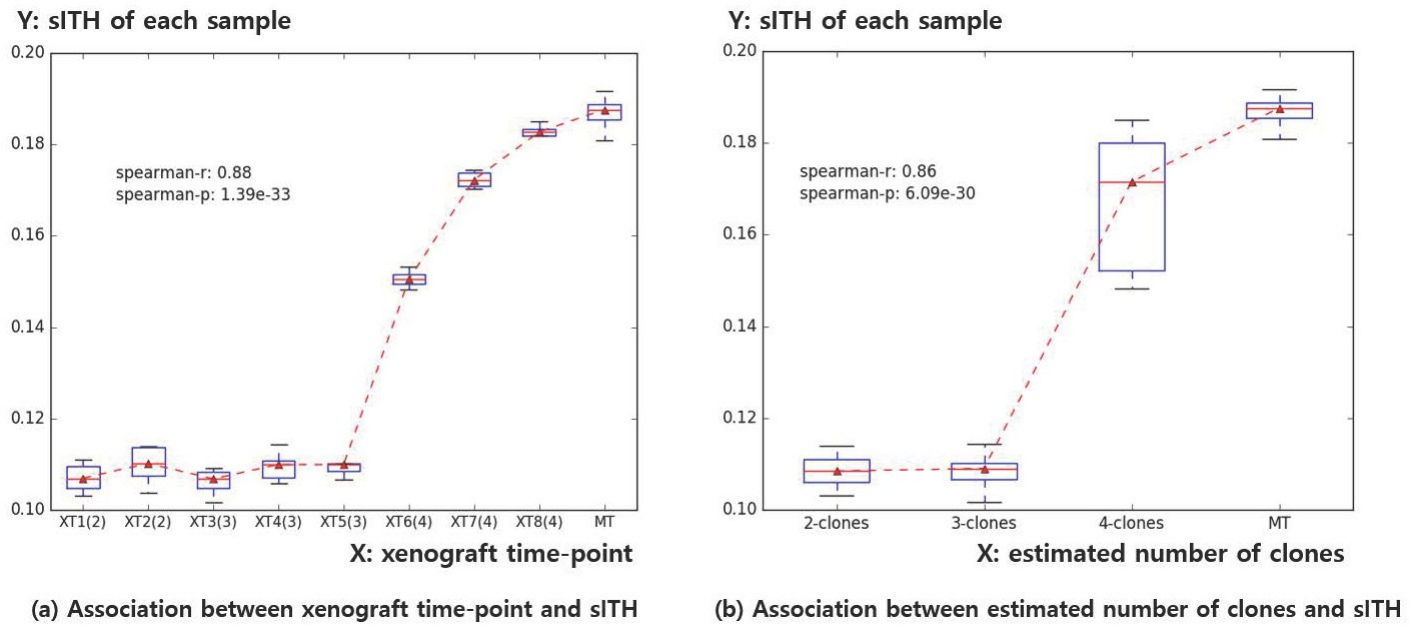
Two boxplots of how the xenograft time-point and estimated subclone numbers are associated with sITH. a) The X-axis represents when each xenograft tumor sample was collected. The Y-axis represents the sITH for each sample (including repeated measurements for 10 normal tissues randomly selected for each tumor sample). b) Same as a) except that the X-axis represents the number of subclones estimated by PyClone.

Spearman correlation showed a positive correlation between sITH and time-point of each sample (r = 0.88, p = 1.39e-33). This is plausible because tumor ITH levels are known to increase with cancer progression [42]. Spearman correlation test results of gITH and sITH measured by genomic data (SNV, somatic mutation) were also correlated for each sample (r = 0.86, p = 6.09e-30). This indicates that sITH has a positive correlation with ITH measured at the genome level. The above results are summarized in Fig 5(a) and 5(b).

### Performance verification using TCGA pan-cancer data

#### Comparison of genomic ITH with sITH

**Fig 6:**
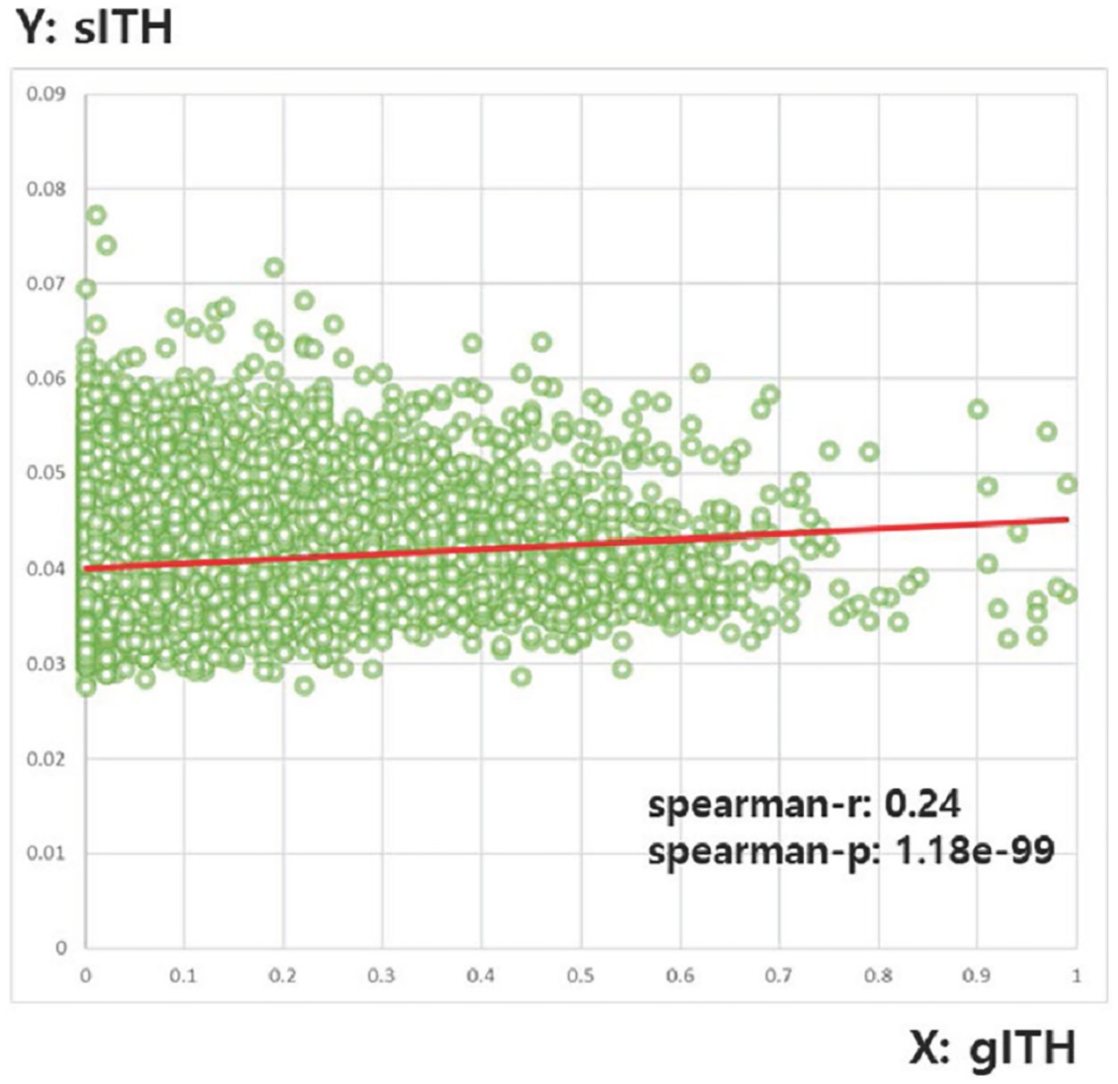
A scatter plot representing the relationship between gITH and sITH.

A study by Weinstein et al. [43] used ABSOLUTE [13] to calculate the gITH of each TCGA pan-cancer sample. Subclonal genome fractions were the gITH we use. Of the 8,297 samples, 698 samples did not have a gITH value, so 7,599 samples were used in this analysis (Table 2). The result shows that sITH and gITH are positively correlated (Spearman: r = 0.24, p = 1.18e-99). The scatter plot between sITH and gITH is shown in Fig 6. gITH is the current gold standard for ITH levels in bulk tumors. Therefore, it was used as a reference standard for sITH in all of the following tests.

Significant but not very large correlations between sITH and gITH suggest that ITH definition at the genome level and ITH definition at the spliceome level may be different. This is a phenomenon also mentioned in other transcriptomic ITH (ie tITH) studies [15], suggesting that heterogeneity levels of the same sample can be estimated differently depending on the type of molecule used in the measurement.

#### Comparison of cancer stage with sITH

**Fig 7:**
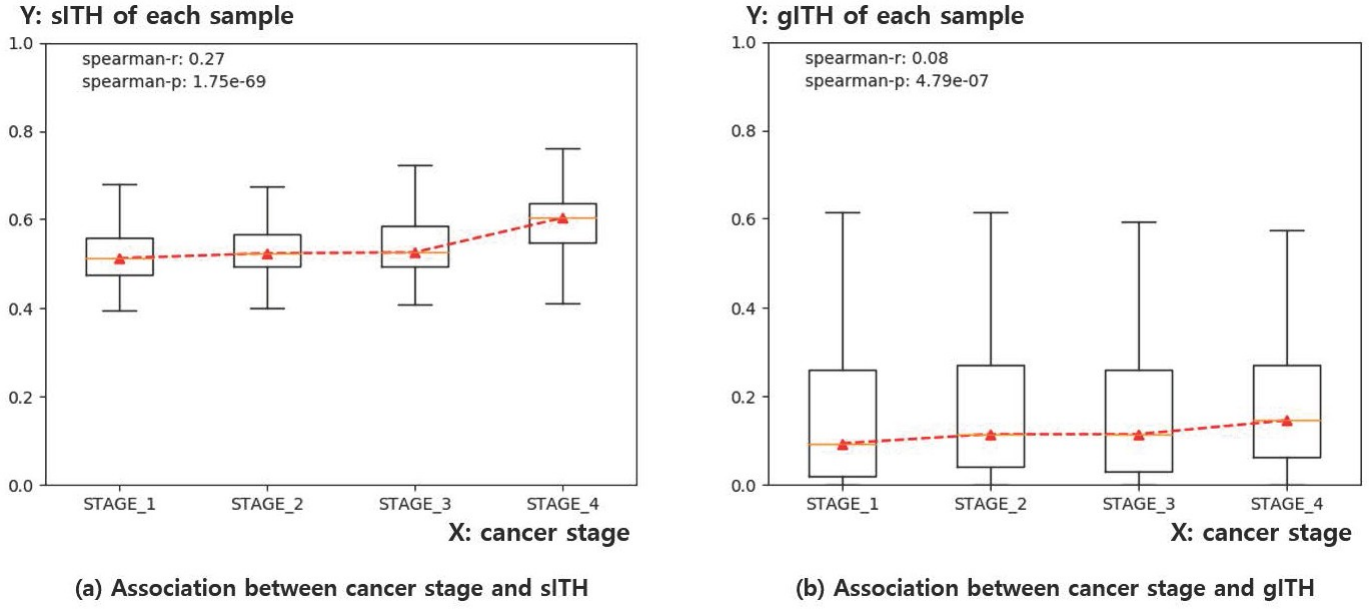
A boxplot showing the association of sITH, gITH and cancer stages in each sample. a) The X-axis represents the cancer stage of each sample (1 to 4 stages). The Y-axis represents the sITH value of each sample. b) Same as a), but in this case, the Y-axis represents the gITH value of each sample. The results show that both sITH and gITH have a significant correlation with the cancer stage, and the significance is greater in sITH. The sITH and gITH values were standardized by dividing the maximum value between samples so that the distribution of the data is easily understood.

The cancer stage is a well-known indicator of cancer progression, which is determined based on pathological observations of cancer tissues such as size, location, the extent of invasion, and extent of spread. The level of ITH is generally related to the progression of cancer. Since 3,363 samples out of 7,599 samples did not have cancer stage information, 4,236 samples were used in this analysis (Table 2). Spearman correlation test results showed that sITH of each sample was positively correlated with cancer stage and sITH showed a better correlation with cancer stage than gITH (gITH: r = 0.08, p = 4.79e-07, sITH: r = 0.27, p = 1.75e-69) (Fig 7).

#### Comparison of survival outcome with sITH

Overall survival represents survival time after treatment, which in this case implies surgical resection of the tumor. The ITH level is associated with the degree of malignancy of cancer and affects the survival rate of cancer patients [10]. So we tested the association between sITH, gITH and the survival outcome of each sample.

**Fig 8:**
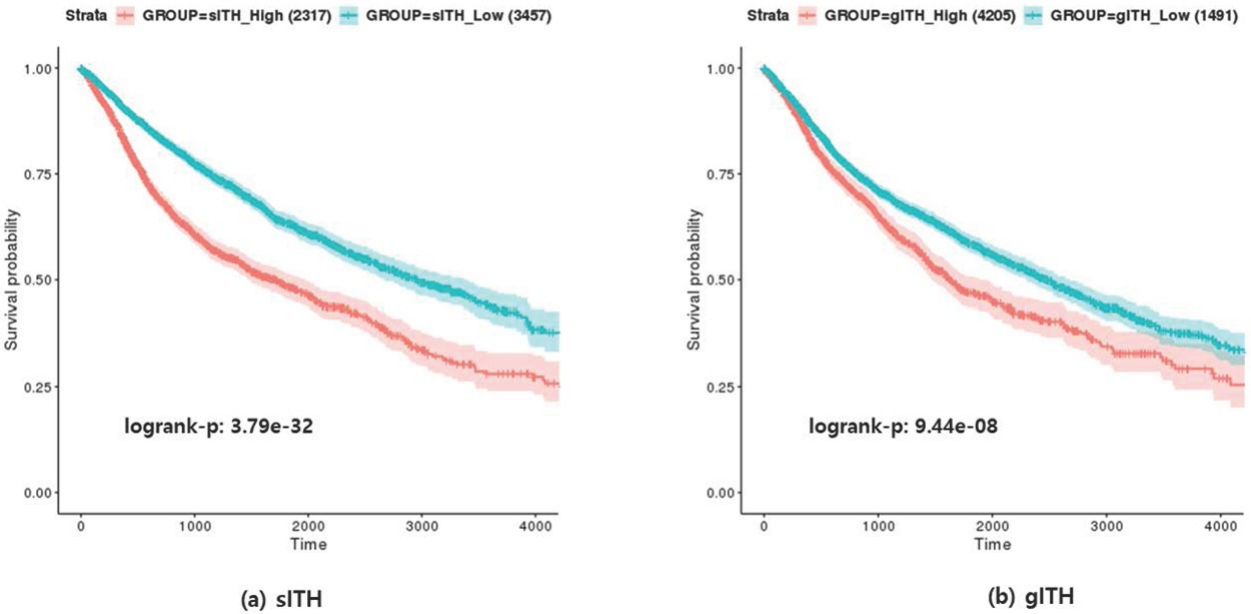
Kaplan-Meier plots of the survival model using sITH and gITH: a) shows the model using sITH, and b) shows the model using gITH.

To test the clinical significance of sITH, we performed a survival outcome association test on 5,265 patient RNA-seq samples. First, the patients were divided into two clusters (scikit-learn python 2.7) with K-means clustering using the sITH value of each sample and log-rank test was performed to test the correlation (p = 3.79e-32) (Fig 8a)). The same analysis was performed using gITH (log-rank p = 9.44e-08) (Fig 8b)). The results of the two experiments show that sITH is a much better indicator of survival outcome than gITH. This difference appears to be due to the different types of molecules on which the two indicators are based, suggesting that it is more effective in predicting prognosis using spliceome than genome data.

We tried to create synergy by modeling the two indicators at once. For each sample, a vector with two variables was used as an input and the same analysis was performed. The log-rank test result was p-value 6.28e-08, which showed better performance than the model using the only gITH, but rather a decrease in performance compared to the model using the only sITH. This means that simply combining the two indicators can not create synergy.

Additional analysis has been prepared to help visual understanding. Initially, 7,599 samples were classified into six groups with different survival outcomes. The first five groups were classified by the time of death. For example, the first group contains samples that died in the first year after treatment, and the second group contains samples that died in the second year. The sixth group includes samples reported to be alive for more than 5 years, where the 5-year threshold is based on criteria commonly used to determine cancer remission. As a result, 2,268 samples were classified into six survival groups and the remainder were excluded because they could not be classified into six groups because of the short follow-up period (Table 2).

**Fig 9:**
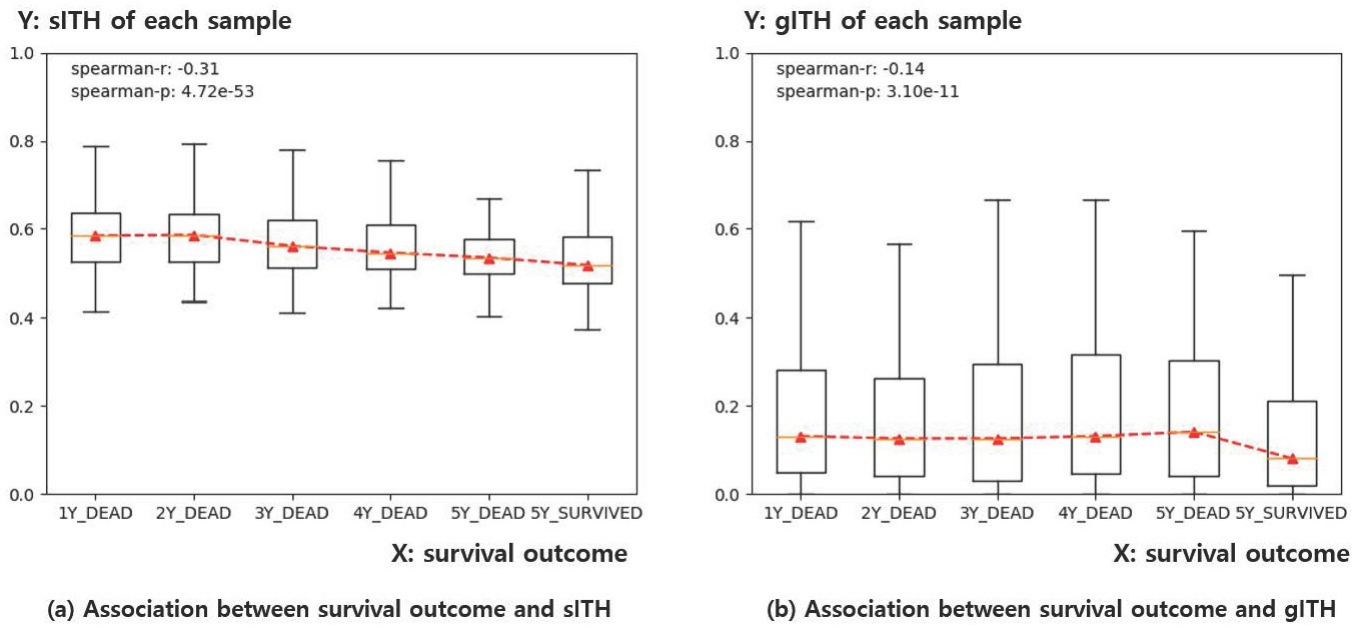
Boxplot for representing the association of sITH and gITH with survival outcome of each sample. a) X-axis indicates the sample group classified by the overall survival outcome (1Y_DEAD 5Y_DEAD, and 5Y_SURVIVAL) of each sample. For example, 1Y_DEAD group indicates the samples dying in 1-year after surgery. Accordingly, 2Y_DEAD corresponds to the samples dying in 2-year (i.e. more than one year, less than two years) after treatment, and so on. Lastly, the 5Y_SURVIVAL group indicates the samples confirmed to survive after 5-year. Y-axis indicates the sITH value of each sample. b) Same as a), except that the Y-axis indicates the gITH value of each sample at this time. Both sITH and gITH had a significant correlation with survival outcome, while sITH showed better association than gITH. Note that sITH and gITH values are standardized by dividing maximum values among samples for easy understanding of the data distribution.

Fig 9 summarizes the association between sITH, gITH and the survival group of each sample. Both gITH and sITH were significantly correlated with survival groups, whereas sITH showed better association (gITH: r = -0.14, p = 3.10e-11, sITH: r = -0.31, p = 4.72e-53). The result indicates that sample groups having higher lethality have a tendency to have greater sITH. The sample information for each sample group is summarized in Table 2.

#### Comparison of PAM50 subtype and sITH

The molecular subtype is derived from molecular information in cancer. One of the most studied cancer type in terms of the molecular level is breast cancer, where the well-known molecular subtype system, PAM50, has been broadly used [44]. PAM50 classifies breast tumors into four types such as Luminal A, Luminal B, Her2-enriched, and Basal-like, where the malignancy of tumor increases as the specified order. First, we performed a Kruskal-Wallis test to determine whether the sITH values among the four subtype groups showed significant distributional differences, which showed a significant difference (p = 5.44e-40). The same analysis was performed with gITH (p = 5.09e-14).

**Fig 10:**
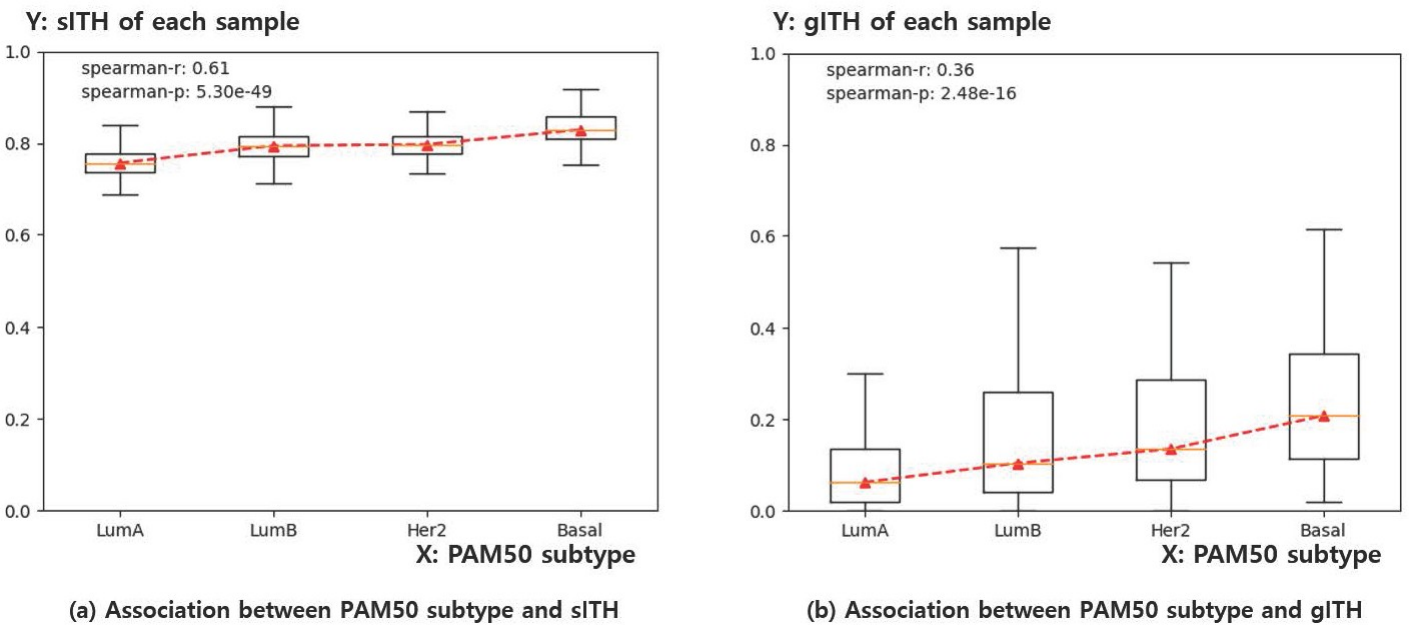
Boxplot for representing the association of sITH and gITH with PAM50 subtype of each breast cancer sample. a) X-axis indicates the PAM50 subtype of each sample, ordered by known malignancy of each subtype. Y-axis indicates the sITH value of each sample. b) Same as a), except that the Y-axis indicates the gITH value of each sample at this time. The result indicates both sITH and gITH have a significant correlation with PAM50 subtype, while sITH shows better association than gITH. Note that sITH and gITH values are standardized by dividing maximum values among samples for easy understanding of the data distribution.

Also, we arranged the samples in the order of Luminal A, Luminal B, Her2-enriched, and Basal-like using the relative malignancy of each subtype. The correlation of the sITH values to the subtype order was then calculated through Spearman correlation. Both ITHs had significant correlation with PAM50 subtype (Fig 10), while sITH showed better association (gITH: r = 0.36, p = 2.48e-16; sITH: r = 0.61, p = 5.30e-49). The result indicates that sample groups expected to have greater malignancy by molecular subtypes tend to have greater sITH. The sample information for each sample group is summarized in Table 2.

## Discussion

The previous tests answered two important questions. The first is whether sITH can measure the ITH of a bulk tumor, and the second is what is the biological and clinical significance of it. The last important question left would be why sITH works, and how does it work. This can be answered using two concepts. One is genetic diversity, and the other is regression toward the mean.

There is no debate that cancer progression and malignization is the product of somatic evolution, whose most important driver is genetic diversity. Genetic diversity is a critical factor in helping tumors survive in various environments, and the ITH is the very indicator of such diversity. As genomic DNA can be used to estimate genetic diversity, so does spliceome. sITH was designed to capture the increase of spliceomic diversity in each intron. The other important factor is the regression toward the mean. Considering the huge differences between cancer patients, We thought that it might not be easy to pinpoint which intronic loci are important in each patient. So we just averaged the entire intronic loci with expecting to capture the average level of genetic diversity for each bulk-tumor. And the results supported our expectation.

Our study suggests that any type of genetic diversities can be used to predict malignancies of bulk-tumors and it also suggests that integrating various sources of genetic diversities might lead to a more comprehensive model of ITH and a better understanding of malignization.

## Conclusion

Despite studies that show intercellular differences at the spliceome level [25, 26], the clinical effect of sITH has not been studied sufficiently because there was no sITH model. SpliceHetero is a sITH model based on local analysis approach that avoids transcriptome assembly which is not easy in cancer RNA-seq. The proposed model has been extensively tested for its performance using synthetic data, xenograft tumor data, and TCGA pan-cancer data. As a result, sITH has shown a strong association with cancer progression and clonal heterogeneity as well as clinically relevant features such as cancer progression, survival outcome, and PAM50 subtype. Also, the distribution of sITH values within each sample group appears more strict than gITH (Figs 7, 9 and 10). That means sITH is a more consistent indicator than gITH.

The proposed model can help to develop diagnostic and prognostic tools by providing a tool to understand the inherent heterogeneity of cancerous spliceome. The whole process is implemented as a software package and is available free at http://biohealth.snu.ac.kr/software/SpliceHetero. It was implemented in Python 2.7 and tested on CentOS Linux release 7 and Ubuntu 16.04, and 18.04. Lastly, note that all the abbreviations used in this article are listed in Table 3.

**Table 3:** List of abbreviations.

ITH	Intratumor Heterogeneity	tITH	Transcriptomic Intratumor Heterogeneity
sITH	Spliceomic Intratumor Heterogeneity	SMRT-seq	Single Molecule Real Time Sequencing
RNA-seq	RNA sequencing	cDNA	Complementary DNA
TCGA	The Cancer Genome Atlas	KLD	Kullback-Leibler Divergence
CNV	Copy Number Variation	NCBI	National Center for Biotechnology Information
WGS	Whole-genome Sequencing	RefSeq	NCBI Reference Sequence Database
gITH	Genomic Intratumor Heterogeneity	KEGG	Kyoto Encyclopedia of Genes and Genomes
SRA	NCBI Sequence Read Archive	JSD	Jensen-Shannon Divergence
NT	Normal Tissue	PT	Primary Tumor Tissue
STAGE	Cancer Stage	SURVIVAL	Survival Outcome
PAM50	PAM50 Breast Cancer Subtyping System	BRCA	Breast Invasive Carcinoma
KIPAN	Pan-kidney Cohort	GBMLGG	Glioma
STES	Esophagus-Stomach Cancers	HNSC	Head and Neck Squamous Cell Carcinoma
LUAD	Lung Adenocarcinoma	LUSC	Lung Squamous Cell Carcinoma
THCA	Thyroid Carcinoma	PRAD	Prostate Adenocarcinoma
BLCA	Bladder Urothelial Carcinoma	COADREAD	Colorectal Adenocarcinoma
LIHC	Liver Hepatocellular Carcinoma	CESC	Cervical Squamous Cell Carcinoma and Endocervical Adenocarcinoma
SARC	Sarcoma	PCPG	Pheochromocytoma and Paraganglioma
PAAD	Pancreatic Adenocarcinoma	UCEC	Uterine Corpus Endometrial Carcinoma
THYM	Thymoma	SKCM	Skin Cutaneous Melanoma
CHOL	Cholangiocarcinoma	OV	Ovarian Serous Cystadenocarcinoma
LAML	Acute Myeloid Leukemia	TGCT	Testicular Germ Cell Tumors
MESO	Mesothelioma	UVM	Uveal Melanoma
ACC	Adrenocortical Carcinoma	UCS	Uterine Carcinosarcoma
DLBC	Lymphoid Neoplasm Diffuse Large B-cell Lymphoma		

## Supporting information

S1 Table The processed sITH values for TCGA pan-cancer dataset. (XLSX)
